# Two novel paralogous *C. elegans* proteins bind to LON-1 but do not have a major role in body size regulation

**DOI:** 10.17912/micropub.biology.002205

**Published:** 2026-06-05

**Authors:** Dillon M. Gardner, Byron Williams, Zhiyu Liu, Jun Liu

**Affiliations:** 1 Department of Molecular Biology and Genetics, Cornell University, Ithaca, NY, United States

## Abstract

The BMP signaling pathway regulates body size and mesoderm fate specification in *C. elegans* . The secreted protein LON-1 both regulates and is regulated by BMP signaling. The mechanism through which LON-1 functions is unclear. In this study, we show that two paralogous and previously uncharacterized proteins, F45D3.3 or F45D3.4 , can bind to LON-1 in vitro. However, removing the two genes does not cause any of the body size or cell fate specification phenotypes exhibited by *lon-1* mutants, suggesting that LON-1 's function in BMP signaling and body size regulation is not primarily through binding to these two proteins.

**Figure 1. Paralogous proteins F45D3.3 and F45D3.4 can bind LON-1 but when knocked out, do not cause BMP-related phenotypes:** **A)** Results of reciprocal co-IP experiments showing that when expressed in Drosophila S2 cells, LON-1 ::V5 can bind to either F45D3.4 ::HA or F45D3.3 ::HA. IP: immunoprecipating. IB: immunoblotting. **B)** The genomic region where F45D3.4 and F45D3.3 reside, with the CRISPR-induced molecular lesions present in the *jj535* allele marked in red below each gene. The start codon of F45D3.4 is marked as 1bp. Arrows mark the locations of the crRNAs used for generating the *jj535* null mutant. **C** ) Predicted F45D3.4 and F45D3.3 protein products encoded by the wild-type (WT) and *jj535* allele, respectively. **D)** Relative body sizes of various strains at the same developmental stage (L4), with WT set to 1.0. NS = no significant difference, *** *P* <0.001 (ANOVA followed by Tukey's honest significant difference (HSD)). **E)** Table displaying the penetrance of the *sma-9 (0)* suppression (Susm) phenotype. Data from *sma-9 ( cc604 )* and *lon-1 ( jj284 ); sma-9 ( cc604 )* were from (Serrano et al., 2025). **** *P* <0.0001, NS = no significant difference (unpaired two-tailed student's *t* test).

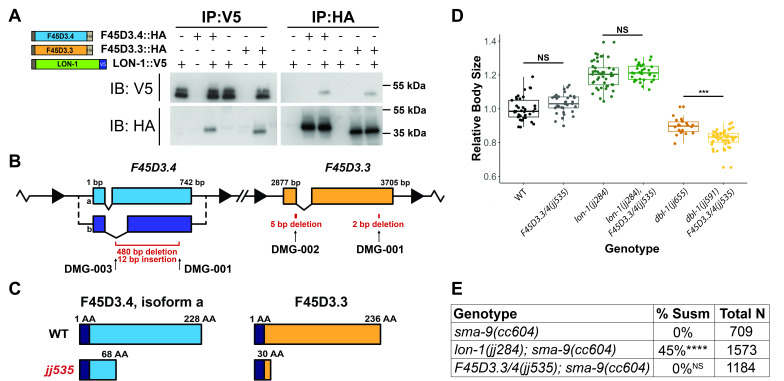



## Description

The highly conserved bone morphogenetic protein (BMP) signaling pathway plays a variety of roles in development and homeostasis across metazoans (Katagiri & Watabe, 2016). In *C. elegans* , BMP signaling is well known to regulate body size: mutations that decrease the strength of signaling yield a shorter worm while mutations that increase the strength of signaling yield a longer worm (Savage-Dunn & Padgett, 2017). BMP signaling also plays a role in fate specification in the *C. elegans* postembryonic mesoderm. Loss-of-function mutations in *sma-9 ,* which encodes a zinc finger-containing transcriptional cofactor for Smad proteins (Liang et al., 2003; Vora et al., 2025), cause the transformation of M lineage-derived coelomocyte cells to sex myoblast cells (Foehr et al., 2006). Mutations in core BMP signaling components as well as regulators of BMP signaling can suppress this loss of M-derived coelomocyte phenotype of *sma-9* mutants, causing a Susm phenotype (Foehr et al., 2006; Liu et al., 2015). We and others have shown that the secreted protein LON-1 both regulates and is regulated by BMP signaling (Liu et al., 2015; Maduzia et al., 2002; Morita et al., 2002). Loss-of-function mutations in the *lon-1* gene result in a long body size (Maduzia et al., 2002; Morita et al., 2002) and a Susm phenotype (Liu et al., 2015). *lon-1* encodes a member of the CAP (cysteine-rich secretory proteins, antigen-5, and pathogenesis-related) super family of proteins, and the CAP domain of LON-1 is capable of binding to sterols in vitro and is required for LON-1 function in vivo (Serrano et al., 2025). However, the precise molecular mechanism of how LON-1 functions to regulate body size and BMP signaling is not fully understood. &nbsp;

To elucidate the mechanism of LON-1 function, we sought to identify LON-1 interactors. Using endogenously tagged sfGFP:: LON-1 (generous gift of Dr. Claire Bénard) and GFP::3xFLAG:: LON-1 as baits, we performed coimmunoprecipitation (co-IP) followed by mass spectrometry (MS) experiments from lysates of these strains using anti-GFP and anti-FLAG antibodies. These experiments identified two proteins, F45D3.3 and F45D3.4 , as the strongest interactors of LON-1 . We verified this interaction by conducting reciprocal co-IP experiments using&nbsp; *Drosophila* &nbsp;S2 cells that overexpress F45D3.3 ::HA or F45D3.4 ::HA and LON-1 ::V5 (Fig 1A). Moreover, in silico structural modeling using Alphafold3 (Abramson et al., 2024) predicted a strong interaction between LON-1 and either F45D3.3 or F45D3.4 (ipTM = 0.65).

The *F45D3.3* and *F45D3.4* genes are located adjacent to each other on chromosome V. The two genes share 69.3% of nucleotide sequence identity and 62.8% of amino acid sequence identity in their coding regions (Madeira et al., 2024), suggesting that they arose from a recent duplication event. Both *F45D3.3* and *F45D3.4* are predicted to encode relatively small proteins (236 aa for F45D3.3 and 228 aa for F45D3.4 ) that each contains a signal peptide ( Fig. 1B, C). Besides the signal peptides, F45D3.3 and F45D3.4 appear to be nematode-specific proteins that do not contain any additional recognizable protein domains.

To determine whether LON-1 regulates body size or BMP signaling through its interaction with F45D3.3 and F45D3.4 , we used CRISPR/Cas9 to generate mutations that simultaneously disrupt both F45D3.3 and F45D3.4 to account for potential functional redundancy shared by the two genes. We isolated one allele, *jj535* , which contains a 480bp deletion that removes most of the coding region of *F45D3.4 ,* as well as two small frame-altering deletions in the coding region of *F45D3.3* , resulting in the truncation of F45D3.3 shortly after the signal peptide ( Fig. 1B, C). Based on the nature of these mutations, the *jj535* allele likely results in the complete loss-of-function of both proteins ( Fig. 1C ). We then measured the body sizes of the *F45D3.3 /4( jj535 )* mutants and found that their body sizes were not significantly different from that of wild-type worms ( Fig. 1D ). We also generated double mutants between *F45D3.3 /4( jj535 )* and *lon-1 ( jj284 )* , a null allele of *lon-1* (Serrano et al., 2025). Again, we found no difference between the body size of *lon-1 ( jj284 )* worms and *lon-1 ( jj284 ); F45D3.3 /4( jj535 )* worms ( Fig. 1D ). These results suggest that F45D3.3 and F45D3.4 do not play a role in regulating body size. Finally, we generated a double mutant between *F45D3.3 /4( jj535 )* and *dbl-1 ,* which encodes the BMP ligand (Mochii et al., 1999; Suzuki et al., 1999). Since both *dbl-1* and *F45D3.3 /4* are located on chromosome V, we generated a *dbl-1* deletion allele *jj591* via CRISPR in the *F45D3.3 /4( jj535 )* background. As a control, we used the same guides and generated a *dbl-1* deletion allele *jj655* in the WT background. As shown in Fig. 1D, *dbl-1 ( jj591 ) F45D3.3 /4( jj535 )* animals are slightly, but statistically significantly smaller than *dbl-1 ( jj655 )* single mutants ( Fig. 1D ). This result suggests that F45D3.3 /4 may have some role in modulating body size, but only in the absence of BMP signaling.

Since *lon-1 (0)* mutants exhibit both a long body size and a Susm phenotype (Serrano et al., 2025), we generated *F45D3.3 /4( jj535 ); sma-9 (0)* compound mutants and asked whether *F45D3.3 /4( jj535 )* displays any Susm phenotype. As shown in Fig. 1E ), *jj535* does not exhibit any Susm phenotype.

In summary, we have found that the CAP domain protein LON-1 can bind in vitro to two previously uncharacterized proteins F45D3.3 and F45D3.4 . These two proteins are novel and nematode specific. They are highly similar and appear to arise due to a recent gene duplication event. We generated an allele that disrupts the genes encoding both proteins and assayed the mutants for their body size and Susm phenotypes, either on their own or in combination with null mutations in *lon-1* or *dbl-1* . Our results suggest that F45D3.3 and F45D3.4 play little or no role in LON-1-mediated body size regulation or mesoderm cell fate specification. Future work will be needed to determine if these two proteins participate in any other BMP-regulated processes.&nbsp;

## Methods

***C. elegans* strains**

All *C. elegans* strains used in this study were maintained using standard culture methods at 20°C. All strains used or generated in this study were derived from the CGC1 reference strain (Ichikawa et al., 2025) or outcrossed into the CGC1 background.

**Immunoprecipitation and mass spectrometry of FLAG- and GFP-tagged LON-1 from *C. elegans***

FLAG tagged LON-1 was immunoprecipitated from worm strain LW4886 using the method exactly as described in (DeGroot et al., 2023) for identifying SMOC-1 interactors. For IP using the GFP tag, total protein from LW4886 and LW7044 strains were extracted as described in (DeGroot et al., 2023) and incubated overnight at 4°C with GFP Trap magnetic agarose beads (Proteintech). For all experiments, CGC1 was used as the negative control. After washing the beads extensively in TBSg10 (20 mM Tris pH 7.6, 150 mM NaCl, 10% glycerol, 0.5 mM EDTA) + 0.1% NP-40, and a final wash using TBS, proteins were eluted using 50 mM Tris, pH 8.0, 2% SDS, and heated for 2 minutes at 95°C. Final eluates from the FLAG and GFP immunoprecipitations were confirmed to contain tagged LON-1 protein by Western blots using anti-FLAG (M2, Sigma) and anti-GFP antibodies (Rockland). All samples were submitted for mass spectrometry analysis using the same method as described in (DeGroot et al., 2023) by the Proteomics and Metabolomics Facility at the Cornell Biotechnology Resource Center.

**Expression and co-IP from Drosophila S2 cells**

Transfection of Drosophila S2 cells and protein collection were performed exactly as previously described (DeGroot et al., 2023). Separate populations of S2 cells were transfected with either F45D3.4 ::HA, F45D3.3 ::HA, or LON-1 ::V5, and the media containing the secreted proteins were collected. Proteins were mixed together and either the anti-HA or anti-V5 beads were used to pull down one or the other protein. Western blot analysis followed. Affinity beads, western blot antibodies, and protein gel techniques were exactly as those used in (DeGroot et al., 2023).

**CRISPR/Cas9 experiments**

The GFP::3xFLAG:: LON-1 allele was generated according to the protocol in (Dickinson et al., 2015). The *jj535 , jj591 ,* and *jj655* alleles were generated according to the protocol outlined in (Ghanta et al., 2021) using guide RNAs specific to the targeted gene(s) with no repair template. crRNAs were selected based on their proximity to the ATG and STOP codons of their targeted genes and their predicted cutting efficiency using the CHOPCHOP tool (Labun et al., 2019). Initial genotyping was conducted through PCR, and allele sequences were obtained through Sanger sequencing.

**Body size analysis**

Worms at the L4.1 and L4.2 larval stages (Mok et al., 2015) were selected from mixed-stage plates and immobilized on a 2% agarose pad on a glass slide in 0.5 mM levamisole in M9 solution. Worms were imaged with a Leica DMRA2 compound microscope equipped with a Hamamatsu Orca-ER camera and the iVision software (Biovision Technology) using a 10X objective lens. Images were opened in Fiji ImageJ, and the segmented line tool was used to measure the length of the worms' midlines, from the tip of the head to the tip of the tail. Statistical analyses were conducted in Rstudio using Tukey's honest significant difference (HSD) test.

***sma-9 (0)* suppression (Susm) assay**

Adult animals were scored for the number of coelomocytes using a secreted coelomocyte GFP marker, *arIs37* (Fares & Greenwald, 2001). Counts reflect the total number of adult worms scored on 3 plates each for 2 independent isolates. Statistical analysis was conducted using unpaired two-tailed student's *t* test.

## Reagents

**Strains:**

**Table:** 

**Strain**	**Genotype**
CGC1	*Caenorhabditis elegans* wild-type (WT)
LW2596	*arIs37 [myo-3p::ssGFP+ dpy-20 (+)] I; cup-5 ( ar465 ) III; sma-9 ( cc604 ) X *
LW4886	*lon-1 ( jj242 [SP::GFP::3xFLAG:: LON-1 ]) III *
LW5758	*lon-1 ( jj284 ) III *
VQ979	*lon-1 ( qv28 [SP::sfGFP:: LON-1 ]) III *
LW7044	*lon-1 ( qv28 [SP::sfGFP:: LON-1 ])* *III* 2xoutcrossed with CGC1
LW7068	*F45D3.3 ( jj535 ) F45D3.4 ( jj535 ) V *
LW7069	*F45D3.3 ( jj535 ) F45D3.4 ( jj535 ) V* 2xoutcrossed isolate #1
LW7070	*F45D3.3 ( jj535 ) F45D3.4 ( jj535 ) V* 2xoutcrossed isolate #2
LW7071	*lon-1 ( jj284 ) III;* *F45D3.3 ( jj535 ) F45D3.4 ( jj535 ) V* isolate #1
LW7072	*lon-1 ( jj284 ) III;* *F45D3.3 ( jj535 ) F45D3.4 ( jj535 ) V* isolate #2
LW7073	*arIs37 [myo-3p::ssGFP+ dpy-20 (+)]) I; cup-5 ( ar465 ) III;* *F45D3.3 ( jj535 ) F45D3.4 ( jj535 ) V; sma-9 ( cc604 ) X isolate #1 *
LW7074	*arIs37 [myo-3p::ssGFP+ dpy-20 (+)]) I; cup-5 ( ar465 ) III;* *F45D3.3 ( jj535 ) F45D3.4 ( jj535 ) V; sma-9 ( cc604 ) X isolate #2 *
LW7440	*dbl-1 ( jj655 ) V *
LW7336	*dbl-1 ( jj591 ) F45D3.3 ( jj535 ) F45D3.4 ( jj535 ) V *

**&nbsp;**

**Oligonucleotides:**

**Table:** 

**Primers used for PCR genotyping**	
*lon-1 ( jj242 ) *	ZL-431 - GGAGAATCTGTACTTTCAATCCGG
**&nbsp;**	ZL-524 - GGGTTACACGTCACGACATATGG
**&nbsp;**	ZL-525 - TATAGGTCTTCAAATACGAAGGTCCC
*lon-1 ( qv28 ) *	ZL-524 - GGGTTACACGTCACGACATATGG
**&nbsp;**	MVS-71 - CCTTCACCCTCTCCACGGAC
**&nbsp;**	JKL-1862 - TAATTTGTCAGTTGAAAAAATTTTAACGACTCTTC
*lon-1 ( jj284 ) *	JKL-1863 - GTGCAAATTTTCGGCAAATTTTTTAGTGACGATTA
**&nbsp;**	JKL-1924 - AACTGCTACATTTGCCAATAATGC
**&nbsp;**	JKL-1925 - TCCAAAGTAGACATCCAATTGTGTTTC
*dbl-1 ( jj591 , jj655 ) *	DMG-020 - CACACCAATGTCTGCTGCTG
**&nbsp;**	DMG-021 - GTGCCTACTGAAACGAGCCC
**&nbsp;**	DMG-022 - GACCCGTGACACATTGCACC
*F45D3.3 /4( jj535 ) *	DMG-008 - AGGTACAATTACAGAAGCAG
**&nbsp;**	DMG-009 - CAATCCAGCTGCCACTTATC
**&nbsp;**	DMG-010 - CTGAAAAACCAGATGGTTAG
**Sequencing primers**	
*lon-1 ( jj242 ) *	ZL-524 - GGGTTACACGTCACGACATATGG
**&nbsp;**	ZL-525 - TATAGGTCTTCAAATACGAAGGTCCC
*F45D3.3 /4( jj535 ) *	DMG-006 - CCATTGTTTCTACTCCATCC
**&nbsp;**	DMG-007 - ACCGAAGCTATATTTAAGGC
**&nbsp;**	DMG-008 - AGGTACAATTACAGAAGCAG
**&nbsp;**	DMG-010 - CTGAAAAACCAGATGGTTAG
**&nbsp;**	DMG-016 - GCCAAGTTCACTTGTCAATC
**CRISPR guide sequences**	
*dbl-1 ( jj591 , jj655 ) *	DMG-018 - CAAACCGCGTCGGGTAGCGT
**&nbsp;**	DMG-019 - CGACGATGGCGACATAGAAG
*F45D3.3 /4( jj535 ) *	DMG-001 - ACGGAGCAGAGCATTCCCAT
**&nbsp;**	DMG-002 - GAGAAGTCTGACGCTACAGA
**&nbsp;**	DMG-003 - GCCCATCTCCCATGGCACCG

&nbsp;

**Plasmids**

**Table:** 

**Plasmid**	**Description**
pZL96.1	sgRNA1 used to generate SP::GFP::3xFLAG:: LON-1
pZL97.2	sgRNA2 used to generate SP::GFP::3xFLAG:: LON-1
pZL130.7	Repair template used to generate SP::GFP::3xFLAG:: LON-1
pJKL1259	Used to express F45D3.4 a::HA in Drosophila S2 cells
pJKL1260	Used to express F45D3.3 ::HA in Drosophila S2 cells
pJKL1200	Used to express LON-1 ::V5 in Drosophila S2 cells
